# Quantification of Trace-Level DNA by Real-Time Whole Genome Amplification

**DOI:** 10.1371/journal.pone.0028661

**Published:** 2011-12-09

**Authors:** Min-Jung Kang, Hannah Yu, Sook-Kyung Kim, Sang-Ryoul Park, Inchul Yang

**Affiliations:** 1 Center for Bio-Analysis, Korea Research Institute of Standards and Science, Daejon, Republic of Korea; 2 Department of Bio-Analytical Science, University of Science and Technology, Daejon, Republic of Korea; Natural History Museum of Denmark, Denmark

## Abstract

Quantification of trace amounts of DNA is a challenge in analytical applications where the concentration of a target DNA is very low or only limited amounts of samples are available for analysis. PCR-based methods including real-time PCR are highly sensitive and widely used for quantification of low-level DNA samples. However, ordinary PCR methods require at least one copy of a specific gene sequence for amplification and may not work for a sub-genomic amount of DNA. We suggest a real-time whole genome amplification method adopting the degenerate oligonucleotide primed PCR (DOP-PCR) for quantification of sub-genomic amounts of DNA. This approach enabled quantification of sub-picogram amounts of DNA independently of their sequences. When the method was applied to the human placental DNA of which amount was accurately determined by inductively coupled plasma-optical emission spectroscopy (ICP-OES), an accurate and stable quantification capability for DNA samples ranging from 80 fg to 8 ng was obtained. In blind tests of laboratory-prepared DNA samples, measurement accuracies of 7.4%, −2.1%, and −13.9% with analytical precisions around 15% were achieved for 400-pg, 4-pg, and 400-fg DNA samples, respectively. A similar quantification capability was also observed for other DNA species from calf, *E. coli*, and lambda phage. Therefore, when provided with an appropriate standard DNA, the suggested real-time DOP-PCR method can be used as a universal method for quantification of trace amounts of DNA.

## Introduction

Quantification of trace amounts of DNA is of special importance in certain analytical applications where the concentration of a target DNA is very low or only limited amounts of samples are available for analysis. Forensic DNA analysis, detection and quantification of pathogenic agents, and quantification of residual DNA impurity in foods and biopharmaceutical products are typical examples [1]–[3]. Due to technical difficulties concerning quantification of trace-level DNA, special guidelines are often suggested to minimize analytical uncertainties and achieve a standard of best practice for the quantification of trace-level DNA [4]. Certain regulatory guidelines also describe acceptable quantities and technical requirements for analysis of contaminating DNA in foods and drugs. The Food and Drug Administration (FDA) guidelines suggest that the acceptable residual amount of host cell DNA in biopharmaceutical drugs should be below 100 pg/dose, while the acceptable limit of host cell DNA allowed by the European Union (EU) is up to 10 ng/dose [5], [6].

Many different methods for quantification of DNA have been developed and applied for specific uses. UV spectrophotometry reading absorbance at 260 nm is the most common laboratory approach for quantification of DNA. The ordinary UV spectrophotometry is considered effective for 5–50 µg/mL DNA while a significantly improved sensitivity of 1 pg/µL DNA by use of a microliter-sample measuring device was reported [7]. However, it is hard to achieve such a high sensitivity in quantification of practical samples containing only trace amounts of DNA since they would be generally not very concentrated to the level of 1 pg/µL. In addition, contamination of nucleotides, RNA, and proteins will significantly interfere with the UV absorbance-based quantification of DNA [8], [9]. Fluorescence-based techniques are also widely used for quantification of DNA. These methods show much higher sensitivity and accuracy compared with UV spectrophotometry for the quantification of DNA [10]. However, the fluorescence-based method was also subject to interferences by contaminants, and was reported to be not effective for quantification of DNA samples lower than 50 pg/mL [9], [11].

Several other methods were developed for a specific purpose regarding the quantification of an extremely low level DNA, especially for quantification of residual host cell DNA in biopharmaceuticals [3], [12]. The hybridization method relies on radio isotopic or chemiluminescent detection of DNA hybridized to random and sequence-specific probes [13], [14]. Another method known as the ‘threshold method’ utilizes antibody-mediated detection and quantification of DNA captured by single-strand binding protein (SSB) [15]. Both the hybridization method and the threshold method are capable of quantifying picogram levels of DNA. These methods are advantageous in that they can quantify DNA in a sequence-independent manner and are applicable to universal DNA species. However, they also have disadvantages involving a relatively long analysis time, labor-intensiveness, and complicated procedures [16].

Another common platform for analysis of a trace amount of DNA is PCR [17], [18]. Due to the extreme sensitivity and simplicity of experimentation, PCR technology has become the first laboratorial choice both for qualitative and quantitative analysis of DNA. Although sequence-specificity is an incomparable merit of PCR technology, it also involves several important limitations with regard to quantitative analysis of DNA. PCR will amplify and quantify only a specific target DNA, and not the whole DNA content. The quantity of the whole DNA content therefore cannot be measured directly by PCR, but could only be estimated indirectly from the quantity of a specific target DNA. The sequence-specificity of PCR also limits the applicability of the method only to DNA samples containing more than one genome-equivalent amounts. The quantification limit by ordinary PCR will then be 3 pg or above for human genomic DNA, although quantification of femtogram amounts of DNA from viruses and bacteria could be relatively easily achieved [19]. New approaches of amplifying multi-copy genes such as rDNA genes and Alu repeats have been applied to overcome the limited sensitivity of ordinary PCR [20], [21]. Substantially improved quantification sensitivities of 1 picogram human DNA and 300 femtogram CHO cell DNA have been reported respectively by PCRs of Alu repeats and rDNA genes [20], [22]. However, those multi-copy PCR methods are not universally applicable to all DNA species due to species-specificities of the target genes. In this regard, we aimed to develop a sensitive and universal method for quantification of trace amounts of DNA which could enable accurate quantification of femtogram levels of DNA independently of their species.

## Results and Discussion

### Optimization of real-time DOP-PCR conditions

A real-time degenerate oligonucleotide primed PCR (DOP-PCR) strategy was designed to achieve a quantitative estimation of trace-level DNA samples. The DOP-PCR strategy enables the whole genome amplification of a DNA sample regardless of its origin and sequence [23]. The DOP-PCR strategy has two potential advantages compared with ordinary PCR methods: its sequence independence, which enables universal applicability of the method for amplification of any arbitrary DNA species, and a potential sensitivity that is not limited by the requirement for one genome-equivalent amount of DNA as a template. Then in theory, the DOP-PCR method could successfully produce amplicons from a sub-genomic amount of DNA even if the sequences and origins of the target DNA are not known. Therefore, we postulated that DOP-PCR combined with the real-time PCR format could enable universal quantification of sub-genomic amounts of target DNA of arbitrary species.

DOP-PCR primers were first optimized specifically for our real-time PCR strategy. Typical DOP-PCR primers are composed of three distinct sequence elements: an anchoring sequence at the 3′ end, a random sequence in the middle, and a tag sequence at the 5′ end. Among the three sequence elements, the anchoring sequence and the random sequence were thought to directly affect the amplification efficiency of DOP-PCR. Therefore, those regions were the main targets for optimization of primers for the quantitative DOP-PCR method. The most important criterion in evaluation of quantification performances from different real-time PCR conditions was the linearity of a standard curve which would be reflected as a regular spacing of amplification profiles of serially diluted standard DNA samples. Another important consideration was the limit of quantification that could be successfully amplified and distinguished from the no template control sample. During optimization of primers, quantification performances of real-time DOP-PCR experiments using different primers were evaluated by two major criteria, linearity of the standard curve generated from serially diluted standard DNA samples and the limit of quantification

**Figure 1:**
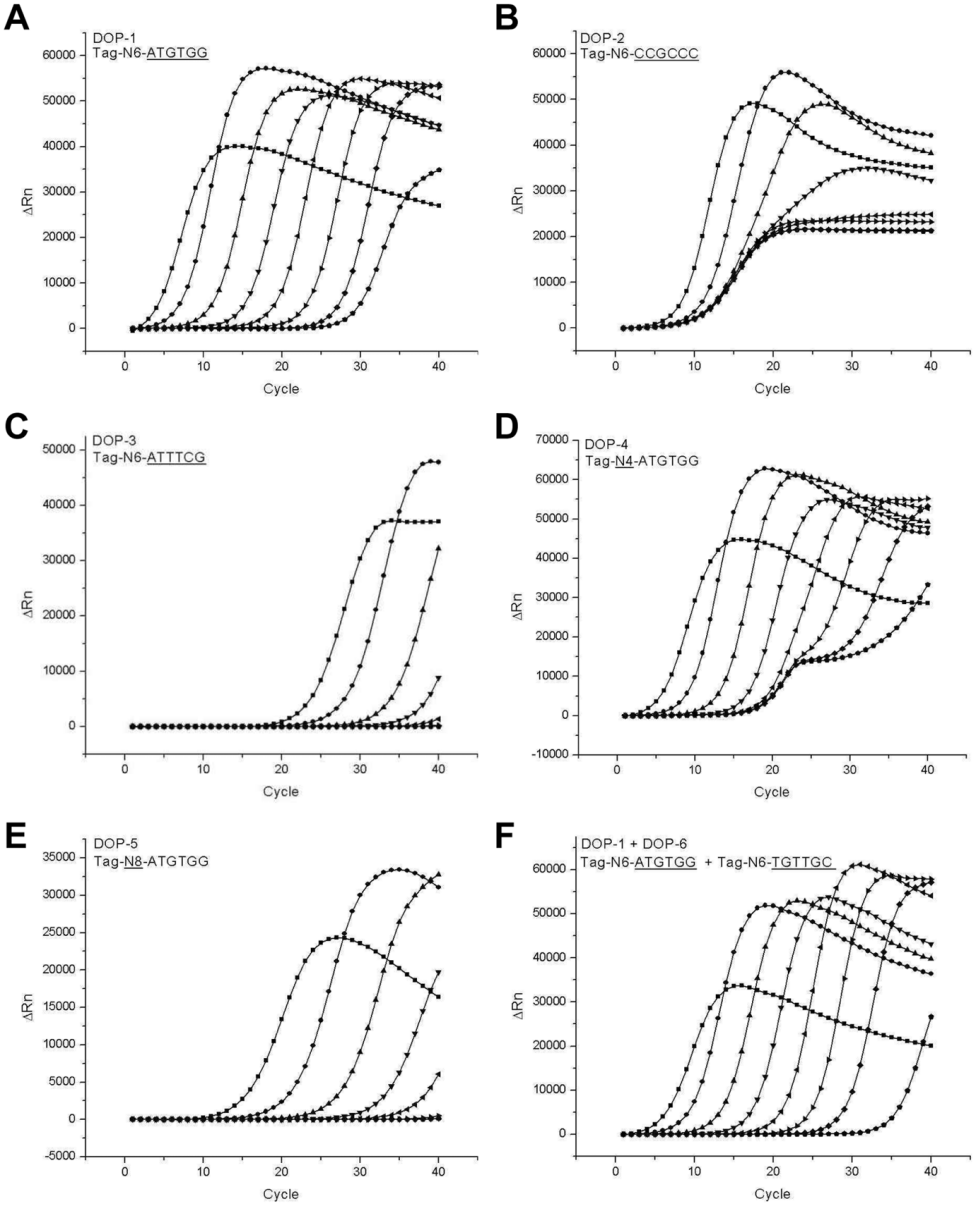
Optimization of primers for real-time DOP-PCR. Amplification profiles of real-time DOP-PCR were obtained by using various degenerate primers. Serially diluted human placental DNA samples ranging from 80 fg to 80 ng and a no-template control (NTC) were amplified. The primers used were Tag-N6-ATGTGG (A), Tag-N6-CCGCCC (B), Tag-N6-ATTTCG (C), Tag-N4-ATGTGG (D), Tag-N8-ATGTGG (E), and a combination of Tag-N6-ATGTGG and Tag-N6-TGTTGC (F).

Amplification profiles obtained from usages of various different degenerate primers are shown in Fig. 1. Amplification profiles from three distinct primers with different GC contents of anchoring sequences were compared (50%, 100%, and 33% GC contents in Fig. 1A, 1B, and 1C, respectively). The primer with a 50% GC content exhibited the best amplification profiles compared to the others. Amplification profiles of even intervals from 8 ng–80 fg DNA standards were obtained using the 50% GC-content primer (Fig. 1A), while poor spacing between amplification profiles (Fig. 1B) or insufficient sensitivity (Fig. 1C) were resulted by using the other primers. The 3′ anchoring sequence determines the frequency, sequence preference, and strength of base pairing in priming of the primers to template DNA. Then, the low GC-content primers will easily dissociate from templates at the elongation temperature of PCR due to weak base pairing. The easy dissociation of primers from templates could result in the decreased initiation and maintenance rate of polymerization, and subsequently lowered amplification efficiency in PCR. Uneven intervals between amplification profiles of serially diluted standard samples obtained from the high GC-content primer seem to be caused by excessive primer dimerization and subsequent nonspecific amplification, which resulted in indiscriminating amplification profiles. Another 50% GC-content primer with a different anchoring sequence of TGTTGC showed similar amplification patterns to those shown in Fig. 1A (data not shown). Therefore, it is apparent that an anchoring sequence with a 50% GC content is the best choice to achieve consistent and sensitive amplification profiles in the real-time DOP-PCR.

The length of the random sequence in the middle of the DOP primer is also an important determinant of DOP-PCR efficiency because it affects the frequency and strength of priming. It was expected that a shorter random sequence in the primer will result in more frequent but lesser strong priming of primers to templates during PCR. Completely opposite results of lesser frequent but stronger priming of primers were expected by using a longer random sequence in the primer. Therefore, the length of the random sequence should also be optimized. Real-time amplification profiles using primers of different random sequences are presented in Fig. 1 (4, 6, and 8 bases in Fig. 1D, 1A, and 1E, respectively). Random sequence of 6 bases (N6) exhibited the best performance showing even intervals and high sensitivity (Fig. 1A), while uneven spacing of amplification profiles (Fig. 1D) and insufficient sensitivity (Fig. 1E) were resulted from the use of 4 bases (N4) and 8 bases (N8) of random sequences, respectively. Based on these results, we concluded that a primer with a 50% GC content in the anchoring sequence and 6 bases of a random sequence in the middle would be the best choice for performing real-time quantitative DOP-PCR. The concentration of the primer in DOP-PCR was also optimized. Use of a lower concentration of the DOP primer resulted in decreased sensitivity while a higher concentration exhibited uneven spacing of amplification profiles (data not shown). It seems that the decreased sensitivity by use of a low-concentrated primer had resulted from the decreased frequency of priming due to insufficiency of primers while disproportional amplification profiles by use of a high-concentrated primer were caused by increased dimer formation and subsequent nonspecific amplification during the DOP-PCR.

It should be noted that the 80-ng sample produced an apparently different amplification profile that did not accord with those of the other standard samples even under the optimized DOP-PCR condition (Fig. 1A). The apparently discordant amplification profile indicated that DNA was amplified under an apparently different amplification kinetics in the 80-ng DNA sample, so that the quantification strategy employed in the current real-time DOP-PCR could not be extended to that level of DNA. Non-negligible levels of fluorescence signals were persistently observed in the no template control (NTC) samples. Those signals might have resulted from an increased rate of primer dimerization owing to random sequences in the primer and subsequent increased non-specific amplification. It could have also resulted from amplification of tiny amounts of contaminating DNA in the PCR reagents, especially in the Taq polymerase. In any case, the limit of the quantification by the optimized real-time DOP-PCR was not further extended below 80 fg, since amplification profiles from 80 fg or lower samples were not distinguishable from that of NTC. It is also noteworthy that a combination of the two best primers (50% GC contents and 6 random sequences) did not produce distinguishably better amplification profiles than those by single best primers (Fig. 1F). Therefore, we used only one primer seen in Fig. 1A for the remaining real-time quantitative DOP-PCR experiments.

### Application of DOP-PCR to different species of DNA

**Figure 2:**
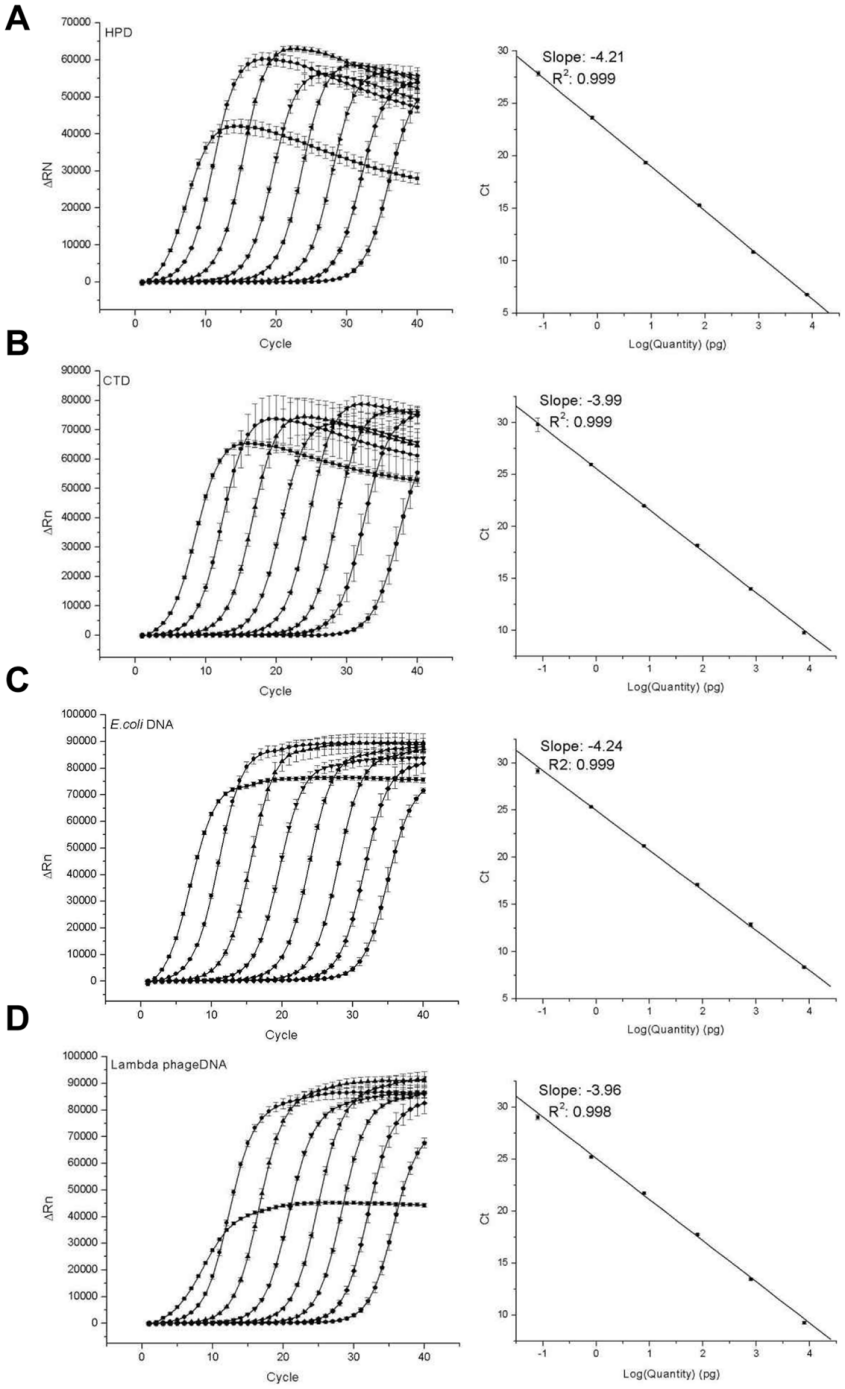
Application of the real-time DOP-PCR to diverse DNA species. Amplification profiles and their standard curves were obtained from human placental DNA (HPD; A), calf thymus DNA (CTD; B), *E. coli* DNA (C), and lambda phage DNA (D). Standard DNA samples from 80 fg to 80 ng and a no-template control were amplified. Six independent experiments each comprising triplicate reactions were performed, and typical results of one experiment are presented. Data for 80 ng and NTC were omitted for the plotting of standard curves.

To assure the general applicability of the method to diverse DNA samples, DNA samples of different origins and different complexities were tested. Amplification profiles and their relevant calibration curves of serially diluted standard DNA samples from human, calf, *E. coli*, and lambda phage are presented in Fig. 2. The figures represent typical examples of amplification profiles and their relevant standard curves from six independent experiments. All four DNA species resulted in the similar patterns and exhibited even intervals between amplification curves of serially diluted DNA standards. All showed successful amplifications of 80-fg templates, which were clearly distinguishable from those of the no-template controls (NTC). However, all amplification profiles of the 80-ng DNA samples regardless of their origins exhibited certain distinctive patterns which did not accord with those of other template amounts. This observation implied that the phenomenon of disproportional amplification of the 80-ng DNA in DOP-PCR was not caused by a sequence-dependent mechanism, but simply by a quantity-dependent mechanism. It is supposed that too high rates of self-annealing between denatured templates and subsequent interferences with normal primer annealing for PCR have led to the discordant amplification patterns in the 80-ng template reactions.

The theoretical basis for quantification of DNA by real-time PCR resides in the assumption that amounts of amplified DNA are proportional to the amounts of template DNA in pre-saturation stages of amplification. Such a proportionality and repeatability of real-time PCR would be represented by a calibration curve calculated from a set of serially-diluted standard DNA samples. Therefore, the validity and accuracy in quantification of DNA by the current real-time DOP-PCR were evaluated by the calibration curves themselves. All standard curves exhibited a good linearity (R^2^ values from 0.995 to 0.999) for diverse DNA species ranging from 80 fg to 8 ng. Data for 80 ng were omitted from the plotting of standard curves since inclusion of those data severely impaired the linearity of standard curves. The good linearity of standard curves strongly supports the conceptual validity concerning quantification of DNA by DOP-PCR for 80-fg to 8-ng DNA samples. Furthermore, the low variability of data not only among triplicate reactions, but also among six independent experiments, indicated the high consistence and reproducibility of the quantitative real-time DOP-PCR. For example, in the analysis of HPD, the standard deviations of threshold cycle (C_t_) values from six independent experiments were 0.30, 0.10, and 0.44 for the 8-ng, 80-pg, and 80-fg DNA samples, respectively (data not shown). Those variabilities in C_t_ values respectively correspond to 17.9% ((10^0.30/4.18^−1)×100%, where 4.18 is the average intervals of C_t_ values between 10-fold diluted DNA samples), 5.7%, and 27.4% of variabilities in assigning DNA quantities based on the standard curve. Then, the measurement uncertainties in quantification of DNA by real-time DOP-PCR are expected to be of those levels. However, the accuracy, the analytical precision from multiplicated reactions in a single experiment, and the measurement uncertainty from multiple independent experiments will also be influenced by various other experimental parameters such as quality of the template DNA, fidelity of PCR instruments, and proficiency of experimenters. Then the practical accuracy and the practical measurement uncertainty could be more or little variable depending on those experimental parameters in quantification of real samples in the fields.

The slope of a standard curve is mathematically correlated to PCR efficiency according to the equation E = 10^−1/slope^−1, where E is the PCR efficiency [24]. A 100% efficiency corresponds to a slope value of −3.32. Slopes of the real-time DOP-PCR experiments ranged from −3.9 to −4.1 depending on the species of template DNA. These slope values correspond to amplification efficiencies of 70 to 80% in DOP-PCR which are lower than those in ordinary real-time PCR. This means that about 20 to 30% of template DNA molecules failed to serve as successful templates to produce new DNA molecules in each PCR cycle. This might have resulted from decreased priming efficiency of perfect match primers to templates by competition with partially complementary primers. Since random sequences are included in the DOP primer, there will exist excessive amounts of partially complementary primers, which could compete and interfere with the perfect match primers for priming. Another possibility is self-annealing of template DNA molecules. In contrast to ordinary PCR, all amplicons have the same primer sequences at the both ends except for random sequences. Therefore, the possibility of self-annealing among denatured template molecules would be greatly elevated in DOP-PCR, which could lead to decreased amplification efficiency. However, in spite of the lower amplification efficiencies of DOP-PCR, standard curves of all DNA species exhibited inter-experimental variations less than 0.5 as C_t_ values that correspond to variabilities about 32% in the calculated amounts of DNA from six independent experiments (data not shown). This suggested that the real-time DOP-PCR is mechanistically stable and consistent so that an accurate and consistent quantification of DNA in a sequence-independent manner could be achieved.

### Determination of absolute DNA quantity

**Figure 3:**
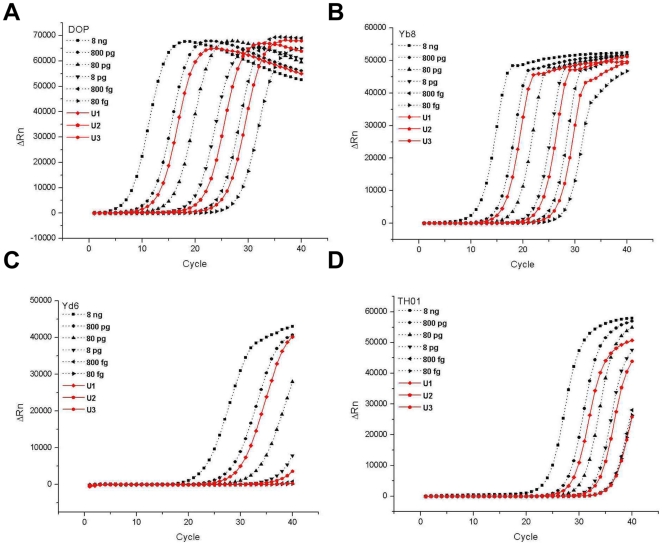
Quantification of laboratory-prepared HPD samples by different real-time PCR approaches. Three laboratory-prepared test samples of HPD were quantified by different real-time PCR approaches: real-time DOP-PCR (A), real-time PCR for the multi-copy Alu, Yb8 (B), the multi-copy Alu, Yd6 (C), and the single-copy TH01 (D). Test samples (solid lines) were amplified in parallel with six standard HPD samples ranging from 80 fg to 8 ng (dotted lines). Gravimetric reference values of the test samples were 403 pg (U1), 4.10 pg (U2) and 418 fg (U3).

To investigate the accuracy and the measurement uncertainty of the real-time DOP-PCR method for quantification of DNA, three laboratory-prepared HPD samples were blind-tested. Several other real-time PCR approaches widely used in biomedical researches and forensic works were also performed in parallel for comparison. They included real-time PCR approaches for the specific single-copy target (TH01) and the primate-specific multi-copy gene, Alu (Yd6 and Yb8). Amplification profiles of three test samples (solid lines) and 80 fg–8 ng standard samples of HPD (dotted lines) are shown in Fig. 3. Real-time PCRs for Yd6 (Fig. 3C) and TH01 (Fig. 3D) exhibited an apparent lack of sensitivity by failing to amplify 800-fg or lower standard DNA samples, while the DOP-PCR (Fig. 3A) and the PCR for Yb8 (Fig. 3B) showed successful amplification of all standard samples. These results were consistent with the previously reported sensitivities of real-time PCR analyses targeting TH01 and Yd6 [22], [25]. It is interesting to note that the sensitivity obtained by real-time PCR of Yb8 was improved to 80 fg in our experiments, while the previous study had reported a sensitivity of 1 pg [22]. The conclusion of Walker *et al.* was based on the observation that although amplification of 100-fg DNA was successful, the amplification profile was not clearly distinguishable from that of the no-template control. Therefore, it is noteworthy that a careful optimization of previously reported PCR conditions could result in a further improved sensitivity. The same sensitivities were observed in amplifications of the test samples. Clear and consistent amplification profiles of all three test samples were obtained by the DOP-PCR and the Yb8-PCR, while indiscrete or no amplification profiles for the test sample of 400 fg (U3) were obtained by the TH01- and Yd6-PCR approaches, respectively.

Estimated quantities of test samples by the real-time PCR approaches were compared with gravimetric reference values (Table 1). As expected from the amplification profiles, all four PCR approaches resulted in satisfactory quantification performances for the test sample of 400 pg (U1). Differences between the measured values and the gravimetric reference values were 7.4% (DOP-PCR), −0.2% (Yb8), 22.2% (Yd6), and 15.6% (TH01). The analytical precisions represented by coefficients of variation (CV) from three independent measurements were 12.6% (DOP-PCR), 1.8% (Yb8), 16.2% (Yd6) and 12.2% (TH01). These results indicated that all four real-time PCR approaches would be similarly adequate for quantification of 400-pg levels of DNA. However, completely different results were obtained for the test samples U2 and U3. Real-time PCR of TH01 and Yd6 resulted in inaccurate estimates or no estimates at all, while the DOP-PCR and the Yb8-PCR exhibited stable and accurate quantification capabilities. Specifically for example of the DOP-PCR, differences between measured values and reference values were −2.1%, and −13.9% with analytical precisions of 15.5%, and 11.3% for the 4-pg and the 400-fg samples, respectively. These values of accuracies and precisions from the DOP-PCR-based measurements were correlated well with the measurement uncertainties predicted by the mathematical analyses of standard curves themselves (5.7–27.4%). Therefore, based on the results, it could be concluded that the real-time DOP-PCR is a stable and accurate method for quantification of DNA ranging from 80 fg to 8 ng. Besides, the real-time PCR for Yb8 also produced very good estimations of the 4-pg and 400-fg DNA samples. Accuracies of −12.9% and −3.1% and precisions of 6.7% and 7.6% were obtained by the Yb8-PCR respectively for the 4-pg and 400-fg DNA samples. They were comparably sensitive and accurate results with those by the DOP-PCR. However, it should be reminded that although the performance of Yb8-PCR was similar with that of the current DOP-PCR method for quantification of sub-picogram amounts of DNA, the Yb8-PCR is of limited applicability only to human and primates DNA.

**Table 1:** Results of blind tests on laboratory-prepared HPD samples.

Sample	Reference*	TH01		Yd6		Yb8		DOP	
		Mean ± SD	Diff. (%)	Mean ± SD	Diff. (%)	Mean ± SD	Diff. (%)	Mean ± SD	Diff. (%)
**U1**	403 (pg)	356±43	−11.6	313±51	−22.2	402±7	−0.3	433±55	7.3
**U2**	4.10 (pg)	7.70±0.20	90.2	2.90±0.51	−28.3	3.50±0.24	−12.9	4.00±0.61	−2.4
**U3**	418 (fg)	ND	ND	ND	ND	387±29	−7.5	360±40	−13.9

* Values obtained from gravimetric dilutions of HPD. SD: standard deviation. ND: not determined.

The overall results presented in this paper confirm that the real-time whole genome amplification method adopting the DOP-PCR strategy is highly stable and accurate for quantification of a wide range of DNA samples from 80 fg to 8 ng. This effective range covers one genome-equivalent amounts of DNA of most mammalians. Therefore, this method would be particularly effective for quantification of a sub-genomic amount of DNA to which ordinary PCR approaches were not generally applicable. Furthermore, the method is universally applicable to a variety of DNA species, regardless of their origins and availability of the sequence information. However, in spite of the universal applicability of the method to various sources of DNA samples, it should be noted that the same species of standard DNA with the target DNA should be used to achieve a highly accurate quantification. Uses of different species of standard DNA could result in significantly lowered quantification accuracy. For example, a 4-pg HPD sample had been quantified as 2.7 pg, 2.5 pg and 1.3 pg by uses of CTD, E. coli DNA and lambda DNA as standards, respectively while an accurate estimate of 3.8 pg was obtained by use of the same species of HPD standard(data not shown).

Based on these results, we suggest the real-time DOP-PCR as a universal method for sensitive and accurate quantification of sub-picogram amounts of DNA. We also believe the method have a potential to be robustly applied in analyses of forensic DNA samples, residual DNA impurities in foods and biopharmaceuticals, and in molecular diagnostics.

## Materials and Methods

### DNA samples

Genomic DNAs from four different species were used as templates for PCR analysis: human placental DNA (HPD, Sigma), calf thymus DNA (CTD, Invitrogen), *E. coli* DNA (extracted from BL21 strain), and lambda phage DNA (NEB). HPD was fragmented by sonication to an average size of 3,000 bp and absolutely quantified by ICP-OES [26]. Calibration standards for real-time PCR were prepared by gravimetric ten-fold serial dilutions of HPD in TE buffer. Test samples for in-house blind tests were prepared also by gravimetric dilutions of HPD. Concentrations of CTD, *E. coli* DNA, and lambda phage DNA were determined by measuring UV absorbance at 260 nm. Absorbance of HPD was measured in parallel to obtain a fit-for-purpose UV extinction coefficient for calculation of concentrations of other DNA species from their measured absorbances. The final values of DNA concentrations were then calculated by applying the obtained fit-for-purpose UV extinction coefficient to their measured absorbances instead of applying the simple equation of 50 µg/mL for an absorbance of 1.

### Real-time PCR

All real-time PCR reactions were performed in triplicate runs. Each reaction mixture was prepared as 50 µL and aliquotted into three 15 µL run-replicates for PCR. Reproducibility of real-time PCR was confirmed by three to six independent experiments prepared and performed on different days. Real-time DOP-PCR was performed in a 15 µL reaction volume containing 80 ng–80 fg of template DNA, 1–2 µM of primers ( 2 µM for single degenerate primer PCR and 1 µM each for double degenerate primer PCR), and the 2X SYBR® premix EX Taq mixture (Takara) using a StepOne™ real-time PCR instrument (Applied Biosystems). Data were collected and analyzed using the built-in StepOne™ Software V2.1. Primers used are described in Table 2. Degenerate oligonucleotide primed PCR (DOP-PCR) was performed following the previously described procedures [23]. In brief, after initial denaturation for 10 minutes at 95°C, five low stringency cycles of 94°C for 60 seconds, 32°C for 90 seconds, ramping to 72°C over a 3-minute period, and 72°C for 180 seconds were performed. Next, forty high stringency cycles of 94°C for 60 seconds, 62°C for 60 seconds and 72°C for 120 seconds were performed. Reactions were completed by final extensions for 7 minutes at 72°C Fluorescence was read only during the high stringency cycles. Primers for DOP-PCR were boiled for 5 minutes at 95°C right before use to eliminate pre-formed primer dimers. Cycling conditions for the TH01 gene or Alu element were as follows: an initial denaturation for 3 minutes at 95°C and 40 cycles of 94°C for 30 seconds, 55°C for 30 seconds, and 72°C for 30 seconds. Primer concentrations for real-time PCR of the TH01 and the Alu element were 0.67 µM.

**Table 2:** Primer sequences.

Primer	Sequence (5′ to 3′)
DOP-1	CCGACTCGAGNNNNNNATGTGG
DOP-2	CCGACTCGAGNNNNNNCCGGCC
DOP-3	CCGACTCGAGNNNNNNATTTCG
DOP-4	CCGACTCGAGNNNNATGTGG
DOP-5	CCGACTCGAGNNNNNNNNATGTGG
DOP-6	CCGACTCGAGNNNNNNTGTTGC
TH01-F	AGGGTATCTGGGCTCTGG
TH01-R	GGCTGAAAAGCTCCCGATTAT
Yb8-F	CGAGGCGGGTGGATCATGAGGT
Yb8-R	TCTGTCGCCCAGGCCGGACT
Yd6-F	GAGATCGAGACCACGGTGAAA
Yd6-R	TTTGAGACGGAGTCTCGTT
